# Editorial: Allo-HSCT: novel clinical applications and therapeutic strategies in adults and analysis of rare procedure complications

**DOI:** 10.3389/fimmu.2026.1904813

**Published:** 2026-07-07

**Authors:** Agnieszka Tomaszewska, Barbara Nasiłowska-Adamska, Luciana Tucunduva

**Affiliations:** 1 Department of Hematology, Transplantation and Internal Medicine, Warsaw Medical University, Warsaw, Poland; 2 Department of Hematology, Institute of Hematology and Transfusion Medicine, Warsaw, Poland; 3 Hematology and Bone Marrow Transplantation, Oncology Center, Sirio-Libanes Hospital, Sao Paulo, Brazil

**Keywords:** allogeneic stem cell transplantation, CRS, GvHD, PT-CY, TA-TMA

Allogeneic hematopoietic stem cell transplantation (allo-HSCT) has an established role as a curative treatment option in patients with different hematologic malignancies, non-malignant disorders and some types of inborn errors of immunity (1). Despite the development of knowledge, improvement of donor selection and therapeutic protocols, this procedure can lead to numerous and sometimes life-threatening post-transplant complications. The major complications after allo-HSCT are graft versus host disease (GvHD), relapse of primary disease, toxicity from conditioning and severe and opportunistic infections (1, 2). Novel therapeutic strategies and applications in the field of allo-HSCT include personalized conditioning, especially in rare diseases or older recipients, innovative prevention strategies of GvHD, novel therapeutic options for certain complications, post-transplant maintenance to prevent relapse and others (1–4).

This Research Topic entitled “Allo-HSCT – novel clinical applications and therapeutic strategies in adults and analysis of rare procedure complications” was intentionally broad in scope and finally has collated 8 manuscripts in this field: 4 original articles, 2 reviews and 2 case reports.

Regarding personalized conditioning regimens, we highlight a prospective investigational study on combination of ruxolitinib and decitabine with modified busulfan-cyclophosphamide (mBu/Cy) for relapse prophylaxis in high-risk acute myeloid leukemia (AML) or myelodysplastic syndrome (MDS) published by Wei et al. (*ClinicalTrials.gov identifier: NCT04582604*). The high risk AML/MDS features were defined as either a relapsed/refractory status, remission status with adverse genetic abnormalities or positive measurable residual disease (MRD+) prior to conditioning. Ruxolitinib (days –15 to –1) and decitabine (days –15 to –10) were administered in 58 recipients followed by mBu/Cy conditioning. The outcomes of a historical cohort of 58 patients (matched 1:1) who received mBu/Cy were described for reference. This study shows interesting results in this very difficult to treat group of patients: the probabilities of 2-year overall survival (OS) – 70.3%, disease free survival (DFS) – 70.6% and graft versus host-relapse-free survival (GRFS) – 65.2%, respectively and 2-year cumulative incidence (CI) of relapse was 19.0%. The main complications were infections: CI of cytomegalovirus (CMV) reactivation – 34.5%, Epstein-Barr virus (EBV) reactivation – 62.1%. Based on their findings, the Authors conclude that this conditioning regimen was safe with a low relapse incidence in these high-risk AML/MDS patients.

Evaluation of pre-transplant factors affecting outcomes after allo-HSCT constitutes a significant issue in transplant procedure planning. There are numerous identified risk factors influencing transplant outcomes, including human leukocyte antigens (HLA) matching, recipient and donor’s age and sex, diagnosis and disease status at transplant, patient comorbidities and many others (1). Additional data in this topic are presented in the retrospective study by Liu et al. utilizing simple and easily accessible parameters like splenomegaly. The Authors demonstrated that the pre-transplant spleen volume obtained by abdominal computed tomography (CT) scans in patients with *de novo* AML was independently associated with poor prognosis after allo-HSCT – affecting mainly OS and NRM non-relapse mortality (NRM).

Graft versus host disease is the main complication after allo-HSCT, leading to significant morbidity and mortality. There are established and standardized protocols for prophylaxis and treatment of GvHD, but steroid-refractoriness or steroid-dependency remains an unmet clinical need in GvHD patients (1, 2, 5). Several methods for second and particularly subsequent lines of therapy have been proposed with inconsistent results. Lu et al. demonstrated promising results of salvage treatment of steroid-refractory hepatic acute GvHD with cyclophosphamide in their retrospective study. This treatment was applied to 50 patients as the second-line or salvage therapy with the overall response (ORR) amounting to 70.0% at day 28 and 66.0% at day 56 and was well-tolerated. The second study on steroid-refractory aGvHD therapy chosen by us in this Research Topic was a metanalysis published by Muhanna et al. The Authors confirmed that extracorporeal photopheresis (ECP) is an effective treatment with favorable response and survival outcomes and is associated with an excellent safety profile. The main disadvantage of ECP is the need of multiple sessions across multiple weeks what constitutes a considerable burden to patients.

Other serious and potentially fatal complications after allo-HSCT are those of endothelial origin, among them thrombotic microangiopathy associated with HSCT (TA-TMA) (1, 6, 7). The pathogenesis of TA-TMA is not fully explained but the proposed “multiple-hit hypothesis” tries to clarify mechanisms leading to this complication (1, 6, 7). The first hit would be the patient’s inherent predisposition to complement activation or pre-existing endothelial injury, the second one endothelial damage from the conditioning regimen and the third hit post-transplant additional factors, including medications (e.g. calcineurin inhibitors - CNI), infections and GvHD. The effectiveness of available therapeutic options of TA-TMA is limited. Major treatment strategies include CNI discontinuation, therapeutic plasma exchange (TPE), TPE plus rituximab, defibrotide and eculizumab (1). Refractory cases of TA-TMA are characterized by multiorgan failure leading to dismal prognosis. New treatments modalities of this serious complication are presented in two case reports published independently by Zhao et al. and by Bao et al. The Authors showed that iptacopan, an oral blocker of complement factor B used in paroxysmal nocturnal hemoglobinuria therapy, can be a valuable and efficacious option in refractory TA-TMA patients.

Another potentially serious complication after allo-HSCT is an early platelet transfusion refractorines (PTR). Retrospective study on an early PTR in 215 patients diagnosed with aplastic anemia was published by Wang Y et al. and showed that it is relatively low frequent complication and not significantly compromise outcomes following successful engraftment in this group of recipients.

Finally, we would like to highlight the review published by Wang et al. dedicated to current understanding and management of cytokine release syndrome (CRS) after allo-HSCT using post-transplant cyclophosphamide (PT-Cy). According to current guidelines, PTCy as a GvHD prophylaxis is extensively used across haploidentical, HLA-mismatched and HLA-matched donor settings (2–4). In general, post-transplant CRS is driven by conditioning-induced tissue injury and donor T-cell alloreactivity and can be life-threatening complication of allo-HSCT (8, 9). The Authors describe in detail pathogenesis and potential management strategies of post-transplant CRS.

In conclusion, this Research Topic article collection highlight the complexity of allo-HSCT procedures, possible early and late complications and their prophylaxis and management. The Guest Editors would like to thank all Authors for their contributions and reviewers for their judicious evaluations.
